# Humans utilize sensory evidence of others’ intended action to make online decisions

**DOI:** 10.1038/s41598-022-12662-y

**Published:** 2022-05-25

**Authors:** Rakshith Lokesh, Seth Sullivan, Jan A. Calalo, Adam Roth, Brenden Swanik, Michael J. Carter, Joshua G. A. Cashaback

**Affiliations:** 1 Department of Biomedical Engineering, University of Delaware, Newark, DE USA; 2 Department of Mechanical Engineering, University of Delaware, Newark, DE USA; 3 Biomechanics and Movements Science Program, University of Delaware, Newark, DE USA; 4 Interdisciplinary Neuroscience Graduate Program, University of Delaware, Newark, DE USA; 5 Department of Kinesiology, McMaster University, Hamilton, ON Canada

**Keywords:** Human behaviour, Decision, Social behaviour, Reward, Dynamical systems

## Abstract

We often acquire sensory information from another person’s actions to make decisions on how to move, such as when walking through a crowded hallway. Past interactive decision-making research has focused on cognitive tasks that did not allow for sensory information exchange between humans prior to a decision. Here, we test the idea that humans accumulate sensory evidence of another person’s intended action to decide their own movement. In a competitive sensorimotor task, we show that humans exploit time to accumulate sensory evidence of another’s intended action and utilize this information to decide how to move. We captured this continuous interactive decision-making behaviour with a drift-diffusion model. Surprisingly, aligned with a ‘paralysis-by-analysis’ phenomenon, we found that humans often waited too long to accumulate sensory evidence and failed to make a decision. Understanding how humans engage in interactive and online decision-making has broad implications that spans sociology, athletics, interactive technology, and economics.

## Introduction

From playing in an orchestra to dancing to competing in sport, our daily lives are enriched by interactions with others. Sensorimotor neuroscience has largely focused on how a single person selects actions. Yet our own evolution, survival, and neural hardwiring have been shaped by our ability to compete and collaborate^1,2^. Following the formalism provided by game-theory in the 1950s^3,4^, research in neuroeconomics and psychology have examined decision-making between humans during collaborative and competitive cognitive-based tasks^5^. Competitive tasks activate different areas of the brain compared to collaborative tasks, suggesting unique neural processes are involved during competitive decision-making^6^. While past work on human interactions has been important to better understand the neural basis of decision-making^7^, it does not consider how the sensorimotor system utilizes online sensory information of another person’s actions to make a decision and select an appropriate motor action.

The neuroeconomics and psychology literatures have used classic game-theory tasks, such as the prisoner’s dilemma and matching pennies, to study cognitive decision-making strategies of interacting humans^3,5,8–10^. In these tasks, the Nash equilibrium solution derived from game theory specifies a decision strategy for each individual, such that no individual gains anything by deviating from that strategy^4,11^. Studies that use cognitive decision-making tasks^10^ and continuous sensorimotor versions of classical games^12^ report that human decision-making behaviour approaches the Nash equilibrium solution. These decision-making scenarios did not account for or consider the potential role of online sensory information of another person’s movement when making a decision. Yet individuals often have online sensory information of another person’s movement that they can utilize to make an informed decision, such as while driving a car^13^ or playing sport^14^. More recently, how two or more humans use bidirectional sensorimotor information to interact has been studied from a continuous sensorimotor control perspective^15–18^ and formally through the framework of optimal feedback control^12,19,20^. These human sensorimotor interaction studies did not examine the role of online sensory information on decision-making.

Decision-making has been studied extensively in the context of an individual animal or human performing sensory discrimination tasks^21,22^. Decision-making research in rodents^23^, non-human primates^24,25^ and humans^26,27^ has linked accumulated sensory evidence and neural activity in prefrontal areas and premotor cortex. Both selected decisions and corresponding reaction time distributions have been well explained with drift-diffusion models^28,29^. Drift-diffusion models rely on accumulating online sensory evidence to appropriately select decisions. Utilizing sensory evidence of another person’s intended action is likely an important feature of interactive human behaviour^30,31^. An unresolved question is how humans utilize online sensory evidence to predict another person’s action and decide how to move.

We designed a two-person, competitive decision-making task where each participant had online sensory information (visual feedback) of their opponent’s actions. Participants received points according to symmetrical or asymmetrical reward structures of the matching pennies game^10^. Across Experiment 1 and 2, we tested the idea that participants accumulate sensory evidence of their opponent’s intended action to make a decision. We manipulated the available trial time to control the amount of sensory evidence available to the participants. We predicted that participants would exploit available time to accumulate sensory evidence when making a decision on how to move. Furthermore, we predicted that the participants would be more likely to move in response to their opponent’s actions with more available time. In Experiment 2, we also tested whether game theoretic predictions for target selection would be preserved when the players shared sensory information about each others’ actions. Accordingly, in addition to modulating available time, here we also manipulated the reward structure experienced by participants. When experiencing an asymmetric reward structure, we predicted that participants would not always select targets in a manner that approached the Nash equilibrium solution since they could utilize sensory evidence of their opponent’s intended action. Finally, we adapted a drift-diffusion model that accumulates sensory evidence to capture online decision-making behaviour of interacting participants.

## Results

### Experimental design

**Figure 1:**
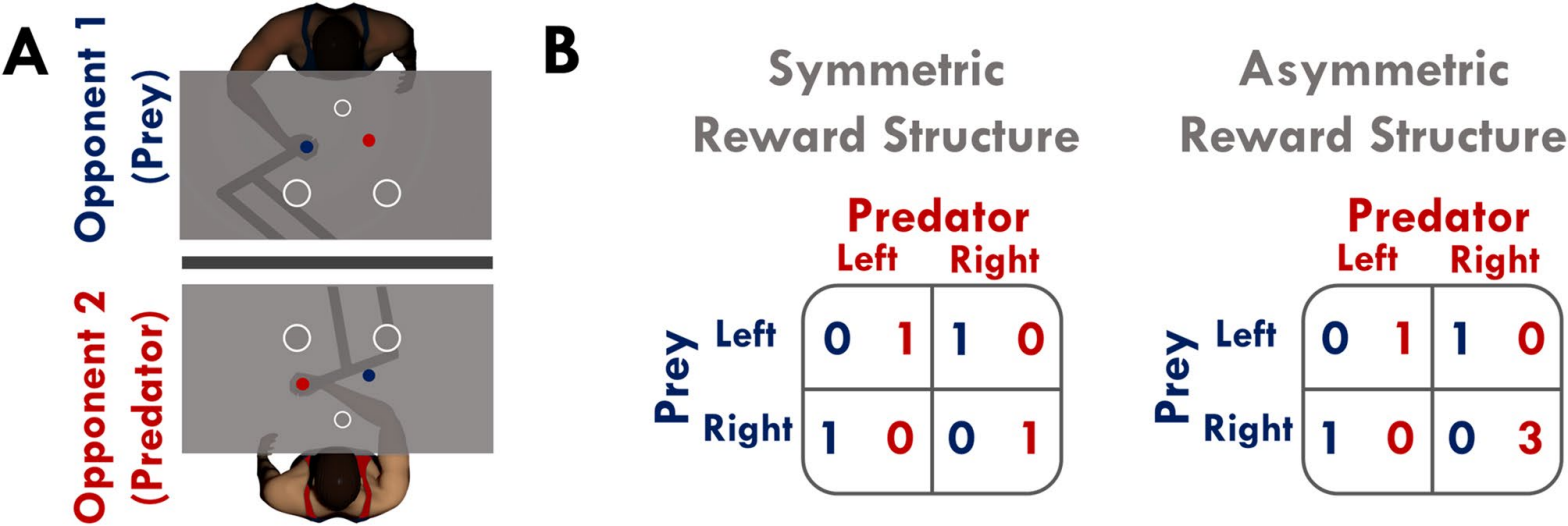
Experiment 1 and 2 design. (**A**) Participants from a human pair each controlled a cursor located at the position of the robot handle. Each participant could see their opponent’s cursor on their respective screens throughout the trial. At the start of each trial, participants placed their cursor in the start position (smaller white ring). Then participants heard a first beep and two targets (larger white rings) would appear. They were instructed to reach forward and move their cursor into one of the two targets before a second beep. Human pairs were randomly assigned to the roles of predator (dark red) and prey (dark blue). The predator won a trial by reaching to the same target as the prey, whereas the prey won a trial by reaching to the opposite target from that of the predator. (**B**) For the symmetric reward structure, the winner of a trial received 1 point and the loser received 0 points. The asymmetric reward structure was similar to the symmetric reward condition, with the exception that the predator received 3 points if both the predator and prey selected the right target. For Experiment 1 and 2, we controlled available time before the second beep (e.g., short = 500 ms, medium = 850 ms, or long = 1500 ms) to manipulate the amount of sensory evidence that a participant could accumulate of their opponent. In Experiment 1, participants experienced three different available times and were rewarded points according to a symmetric reward structure, resulting in the following conditions: short-symmetric, medium-symmetric, and long-symmetric. For Experiment 2, we adopted the two different reward structures (symmetric and asymmetric) to also test whether participants would select targets in proportions that approached the Nash equilibrium solution when they had sensory information of their opponent. Here participants experienced two different trial times (short or long) and two reward structures (symmetric or asymmetric), resulting in the following conditions: short-symmetric, short-asymmetric, long-symmetric, and long-asymmetric. For Experiment 1 and 2, we predicted that participants: (1) would exploit available time to accumulate sensory evidence, and (2) utilize sensory evidence and accordingly move in response to their opponent’s actions. In Experiment 2, we predicted that participants would not select targets in proportions that approached the Nash equilibrium solution in the long-asymmetric condition.

Here we designed a two-player sensorimotor task where participants had visual feedback of their opponent (Fig. 1A). Each participant controlled a visible cursor that was aligned with their hand position. They could also see the online position of their opponent’s cursor. Both participants were instructed to move from a start position and to select one of two potential targets before the end of the trial. The participants were allowed to leave the start position once they heard the first beep and were required to reach a target before a second beep. Once a participant entered a target, their cursor would remain stationary at the target entry point until the end of the trial. For Experiments 1 and 2, participants received points according to the matching pennies game^3^.

In Experiment 1, we tested how humans make informed decisions that rely on sensory evidence of another person’s actions. To manipulate the amount of sensory evidence, we used three blocks of trials with different amounts of available time: short (500 ms), medium (850 ms) and long (1500 ms). Participants were rewarded points according to the symmetric matching pennies game (Fig. 1B) and were assigned fixed roles as either the ‘predator’ or ‘prey’. The predator received one point by reaching the same target as the prey. Conversely, the prey won a point by reaching a target different from the predator. Participants did not receive any points if they did not reach a target before the second beep. Additionally, a participant won the trial if they selected a target and their opponent did not select a target prior to the second beep. Accordingly, there were three conditions: short-symmetric, medium-symmetric, and long-symmetric. Participants completed 150 trials in each condition. We used a 3 (available time: short, medium, long) $\times$ 2 (role: predator, prey) mixed ANOVA to test for main effects and interactions separately for each dependent variable.

In Experiment 2, we further tested how humans make decisions that rely on sensory evidence of another person’s actions. We also tested whether participants would approach game theoretic predictions for target selection (Eq. 3, 4) when utilizing sensory evidence of their opponent’s actions. Participants performed short (500 ms) and long (1500 ms) trial lengths with either a symmetric or asymmetric reward structure (Fig. 1B). The only difference between the asymmetric and symmetric reward structure was that the predator received 3 points if the predator and prey reached the right target. For the symmetric reward structure, the Nash equilibrium solution suggests that both participants should select the right target in a 50% proportion. For the asymmetric reward structure, the Nash equilibrium solution suggests that the predator and prey should respectively select the right target in 50% and 25% proportions. Accordingly, there were four conditions: short-symmetric, short-asymmetric, long-symmetric, and long-asymmetric. Participants completed 150 trials in each condition. We used a 2 (available time: short, long) $\times$ 2 (reward condition: symmetric, asymmetric) $\times$ 2 (role: predator, prey) mixed ANOVA to test for main effects and interactions separately for each dependent variable.

**Figure 2:**
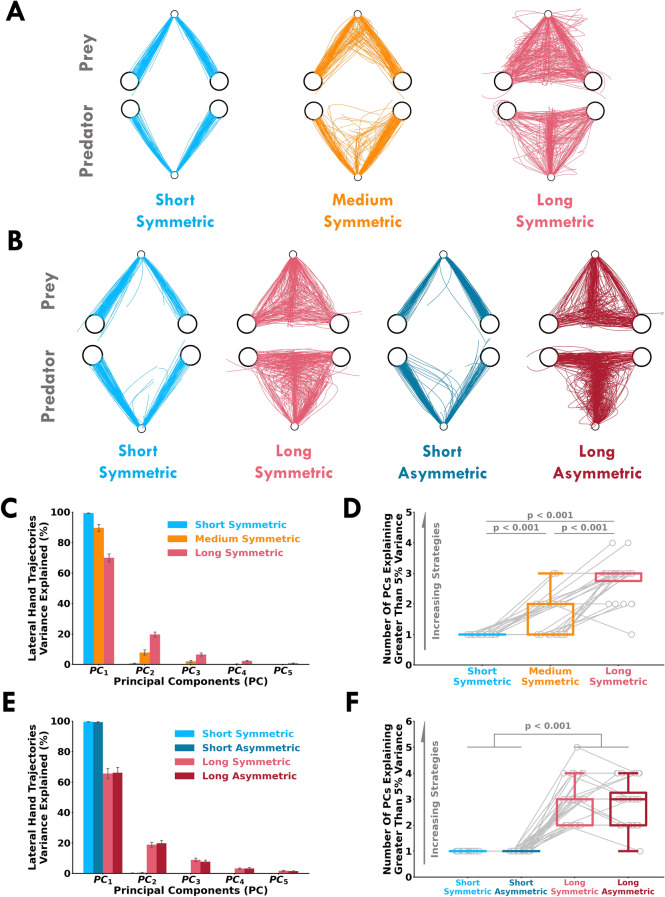
Individual and group movement behaviour. Sample hand trajectories of an exemplar human pair (predator top row, prey bottom row) for all trials within each condition for (**A**) Experiment 1 and (**B**) Experiment 2. Lateral hand trajectory variance explained (*y-axis*) by the first five principal components (*x-axis*) for (**C**) Experiment 1 and (**E**) Experiment 2. Error bars represent $\pm 1$ standard error. Number of principal components explaining greater than 5% variance (*y-axis*) within each condition (*y-axis*) for (**D**) Experiment 1 and (**F**) Experiment 2. The open gray circles and connecting gray lines correspond to individual participants. Box plots show 25th, 50th, and 75th percentiles. Here we see an increase in movement complexity with more available time, which suggests participants used an increased number of strategies to mislead and react to an opponent.

### Trial level movement behaviour

With more available time, participants displayed increasingly more complex reaching trajectories in the medium and long conditions (Fig. 2A,B). An increase in complexity suggests that participants used a broader array of strategies to react to or mislead their opponent. For short trial conditions, participants did not have much available time and reached directly to one of the two targets. As a proxy of the number of movement strategies adopted by the participants, we quantified the complexity of lateral hand trajectories using principal component analysis (PCA) for Experiment 1 (Fig. 2C) and Experiment 2 (Fig. 2E). We included trials where participants reached a target. For each participant and condition, we counted the number of principal components that explained more than 5% of the lateral hand trajectory variance (Fig. 2D,F). The number of principal components (PC) increased with more available time (*p* < 0.001, ${\hat{\theta }}$ > 95.83% for all comparisons). A larger number of PCs suggests that participants used more strategies with more available time.

### Participants exploited available time

**Figure 3:**
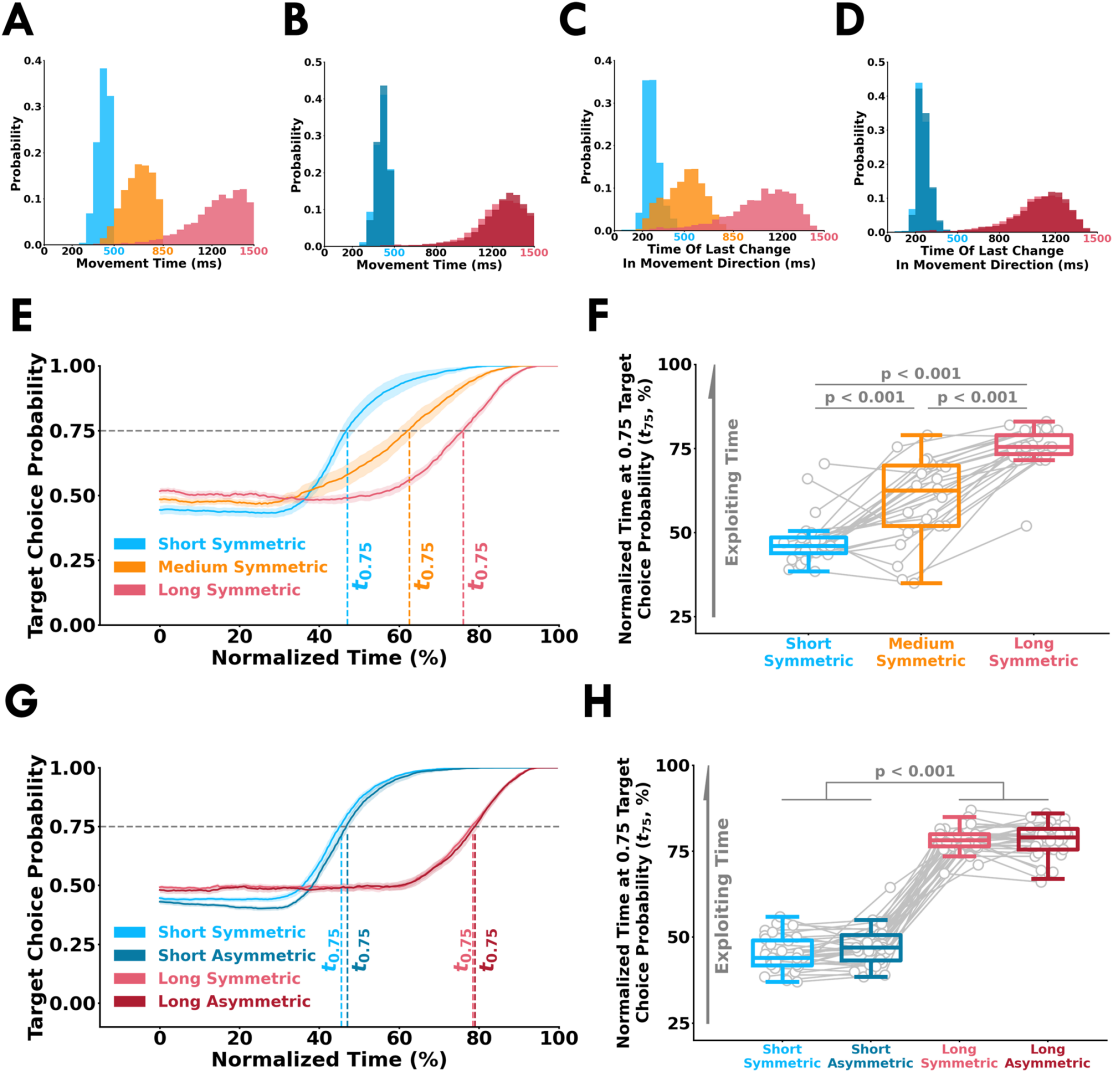
Target selection timing and choice behavior. Movement time (*x-axis*) and probability (*y-axis*) from all participants within each condition for (**A**) Experiment 1 and (**B**) Experiment 2. Time of last change in movement direction (*x-axis*) and probability (*y-axis*) for (**C**) Experiment 1 and (**D**) Experiment 2. Time of last change in movement direction represents when a participant made their final movement towards a target. *Target choice probability* (*y-axis*) across normalized time (*x-axis*) for (**E**) Experiment 1 and (**F**) Experiment 2. *Target choice probability* is the probability that a participant reaches to the right or the left target given the cursor is respectively on the right side or the left side of the workspace. $t_{0.75}$ times indicate the normalized time when the *target choice probability* crosses 75%. Summary of $t_{0.75}$ times (*y-axis*) for (**G**) Experiment 1 and (**H**) Experiment 2. The open gray circles and connecting gray lines correspond to individual participants. Box plots show 25th, 50th, and 75th percentiles. These results align with the notion that participants exploited available time to accumulate sensory evidence of their opponent’s intended action.

Movement times (Fig. 3A,B), and time of last change in movement direction (Fig. 3C,D) from all participants in Experiment 1 and 2. Here, time of last change in movement direction represents when a participant initiated their last movement towards a target. Additionally, we quantified whether participants were exploiting available time by calculating their *target choice probability*. *Target choice probability* is the probability of a participant’s cursor being on the same side (left or right) of the workspace as the eventually selected target. We calculated *target choice probability* along normalized time for Experiment 1 (Fig. 3E) and Experiment 2 (Fig. 3G). We then examined the moment in normalized time that the *target choice probability* crossed 75% ($t_{0.75}$). For $t_{0.75}$ times, we found a significant main effect of trial length in Experiment 1 (F[2, 44] = 63.17, *p* < 0.001) and Experiment 2 (F[1, 30] = 1441.69, *p* < 0.001). As shown in Fig. 3F,H, $t_{0.75}$ times were significantly greater with an increase in trial time (*p* < 0.001, ${\hat{\theta }}$ > 75.00%, for all comparisons). These results indicate that participants exploited available time in the trial by remaining unpredictable about their target selection.

### Participants moved in response to their opponent’s actions with more available time

**Figure 4:**
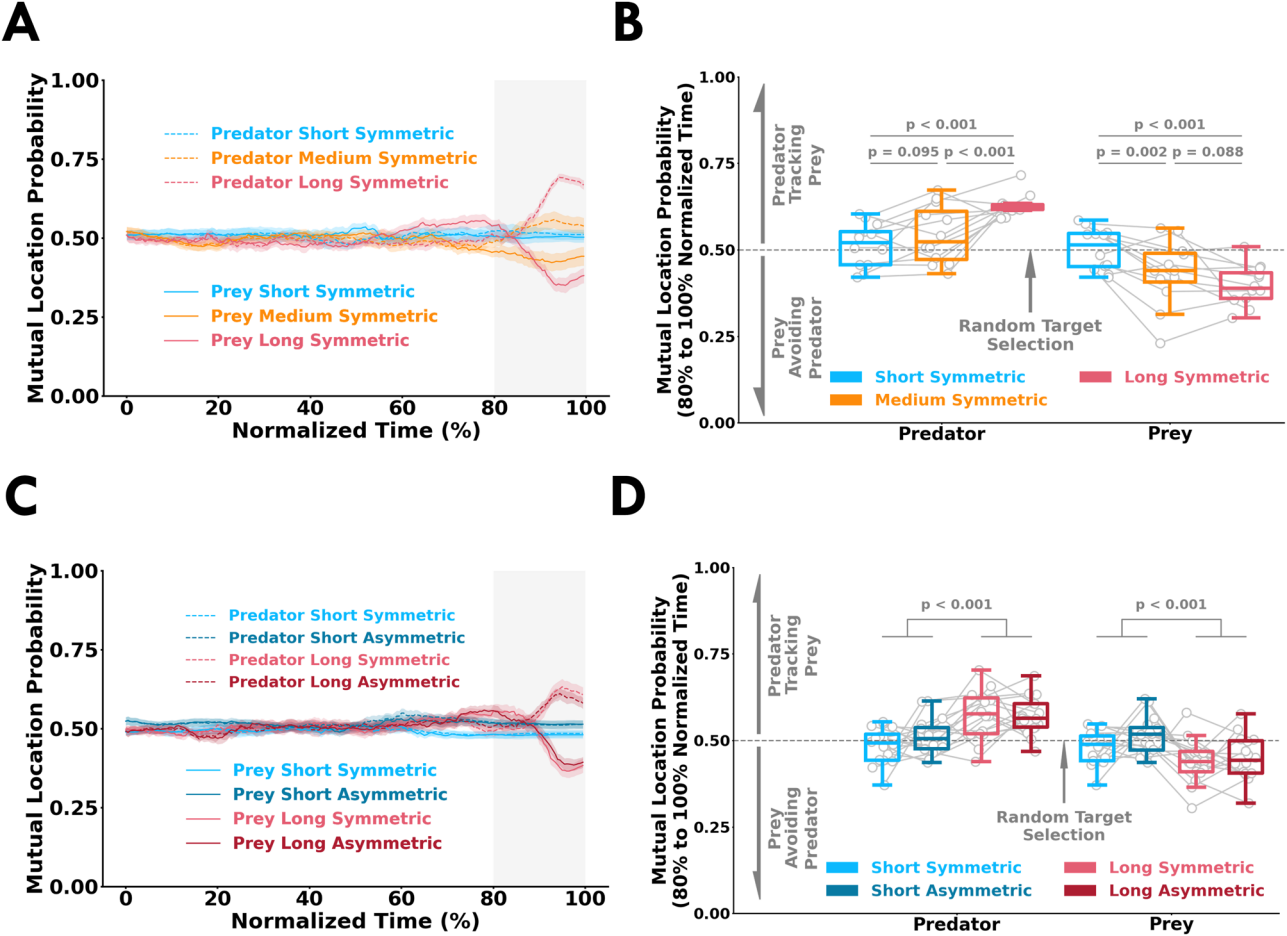
Tracking and avoidance behaviour. *Mutual location probability* (*y-axis*) across normalized time (*x-axis*) for (**A**) Experiment 1 and (**C**) Experiment 2. Average *mutual location probability* in the 80–100% normalized time window (*y-axis*) for each condition in (**B**) Experiment 1 and (**D**) Experiment 2. *Mutual location probability* greater than 0.5 or less than 0.5 represents net tracking or avoidance behaviour, respectively. *Mutual location probability* equal to 0.5 corresponds to random target selection (gray dashed line) and not relying on sensory evidence of an opponent’s movement. The open gray circles and connecting gray lines correspond to individual participants. Box plots show 25th, 50th, and 75th percentiles. Participants did not move in response to their opponent’s movements in the short trial conditions because they had insufficient time to utilize sensory evidence. With more available, the predator and prey respectively exhibited tracking and avoidance behaviour. Thus, with more available time, participants relied on sensory evidence of their opponent’s actions before selecting a target.

We calculated a metric termed *mutual location probability* to quantify whether a participant’s movements were based on observing their opponent. *Mutual location probability* is the probability that the cursors of the predator and prey were on the same side (left or right) of the workspace. A *mutual location probability* greater than 0.5 suggests that the participant was exhibiting tracking behaviour. Conversely, values less than 0.5 suggests that the participant was exhibiting avoidance behaviour. We plotted the *mutual location probability* along normalized time for Experiment 1 (Fig. 4A) and Experiment 2 (Fig. 4C). Here, we normalized time from the start of the trial to the time when the participant entered a target. In the short conditions, the values were always close to 0.5 indicating that the participants did not move in response to their opponent. In the medium and long condition trials, the predator increasingly exhibited tracking behaviour as a trial progressed. Conversely, the prey displayed avoidance behaviour. To assess tracking and avoidance behaviour we looked at the average *mutual location probability* between 80-100% of normalized time for Experiment 1 (Fig. 4B) and Experiment 2 (Fig. 4D). We found a significant interaction between participant’s role (predator or prey) and trial length in Experiment 1 (F[2, 44] = 27.86, *p* < 0.001) and Experiment 2 (F[1, 30] = 49.66, *p* < 0.001). In Experiment 1, the predator displayed significantly greater tracking behaviour in the long-symmetric compared to medium-symmetric (*p* < 0.001, ${\hat{\theta }}$ = 83.33%) and short-symmetric (*p* < 0.001, ${\hat{\theta }}$ = 91.66%). The prey displayed significantly greater avoidance behaviour in the medium-symmetric condition (*p* = 0.002, ${\hat{\theta }}$ = 83.33%) and long-symmetric condition (*p* < 0.001, ${\hat{\theta }}$ = 100%) compared to the short-symmetric condition. Similarly, in Experiment 2, the predator displayed greater tracking behaviour in the long conditions compared to the short conditions (*p* < 0.001, ${\hat{\theta }}$ = 84.37%). The prey had more avoidance behaviour in the long conditions compared to the short conditions (*p* < 0.001, ${\hat{\theta }}$ = 81.25%). For both experiments, the predator and prey used a random target selection strategy (mutual location probability $\approx$ 0.5) that did not rely on observing their opponent in the short conditions (*p* > 0.1, ${\hat{\theta }}$ < 60.00% for all comparisons). Thus, the predator and prey clearly exhibit tracking and avoidance behaviour before reaching a target in accordance to their roles. Differences in mutual location probability between the predator and prey would in part indicate that participants utilize sensory evidence of their opponent’s target selection. We also performed a cross-correlation analysis between the x-coordinate velocities of the predator and prey to further examine tracking and avoidance behaviour. The cross-correlation analysis yielded similar results and the same interpretation as *mutual location probability* (see Supplementary A). The observed tracking and avoidance behaviour suggests that with more available time, participants increasingly relied on the sensory evidence of their opponent’s cursor before selecting a target.

### Correlates of successful performance

**Figure 5:**
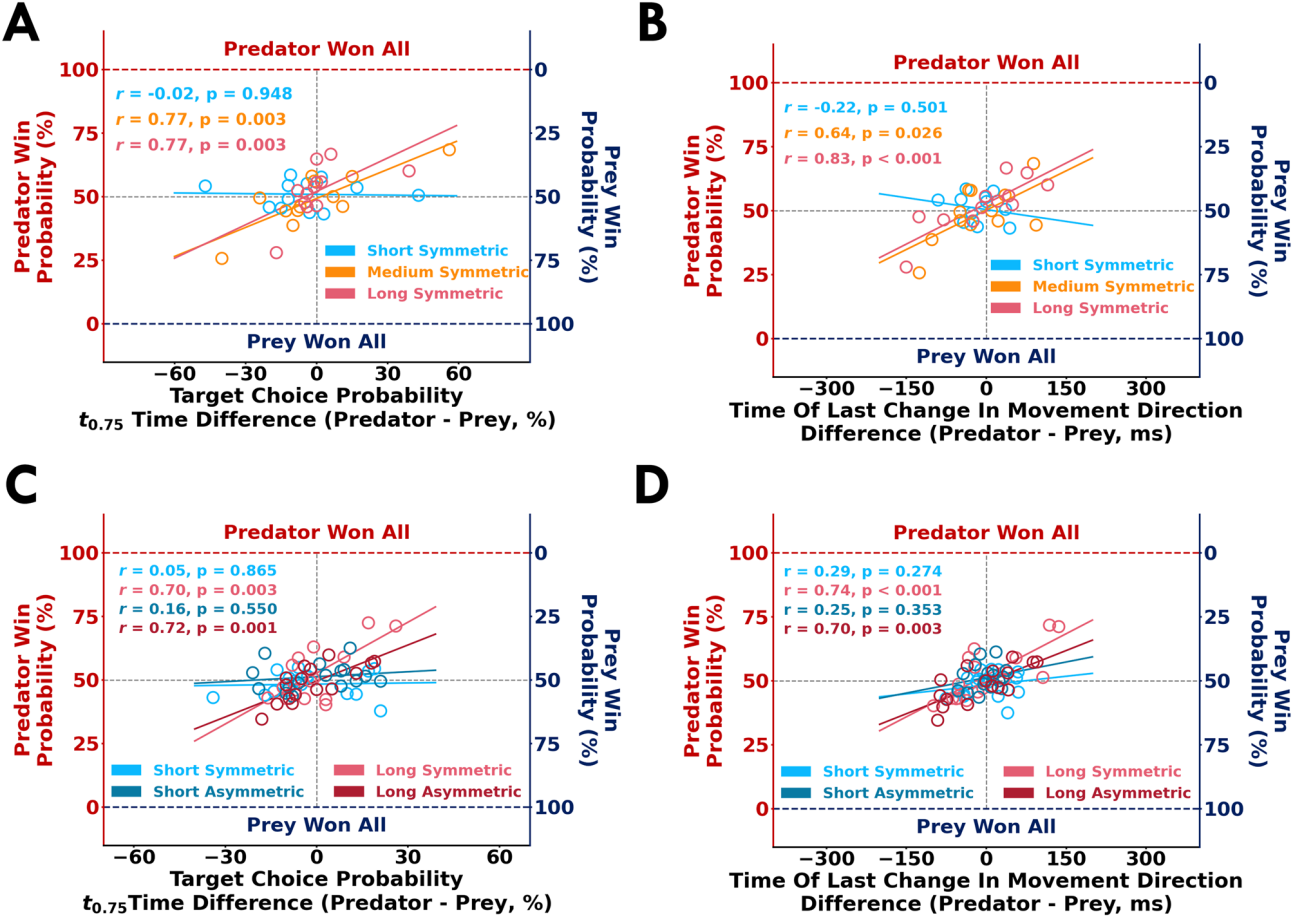
Correlates of successful performance. Relationship between *target choice probability* $t_{0.75}$ time differences (*x-axis*) of the predator and prey to the win probability (*y-axis*) in (**A**) Experiment 1 and (**C**) Experiment 2. Relationship between time of last change in movement direction differences (*x-axis*) of the predator and prey to win probability (*y-axis*) in (**B**) Experiment 1 and (**D**) Experiment 2. Open circles represent pairs of competing participants. Least square regression lines are shown for each condition. With more available time, these data suggest the participants that exploited time and delayed their final reach had a greater win probability.

We were also interested in quantifying behaviour that resulted in successful performance. Across both experiments, participants with a greater $t_{0.75}$ time relative to their opponent had a greater win probability in the medium and long conditions (*p* $\le$ 0.001, r $\ge$ 0.70, for all comparisons; Fig. 5A,C). Further, participants that delayed the *time of last change in movement direction* had a greater win probability in the longer trial conditions (*p* < 0.05, r $\ge$ 0.64, for all comparisons; Fig. 5B,D). These results suggest that successful participants exploited time and delayed their final reach.

### Participants selected targets in proportions that approached Nash equilibrium

**Figure 6:**
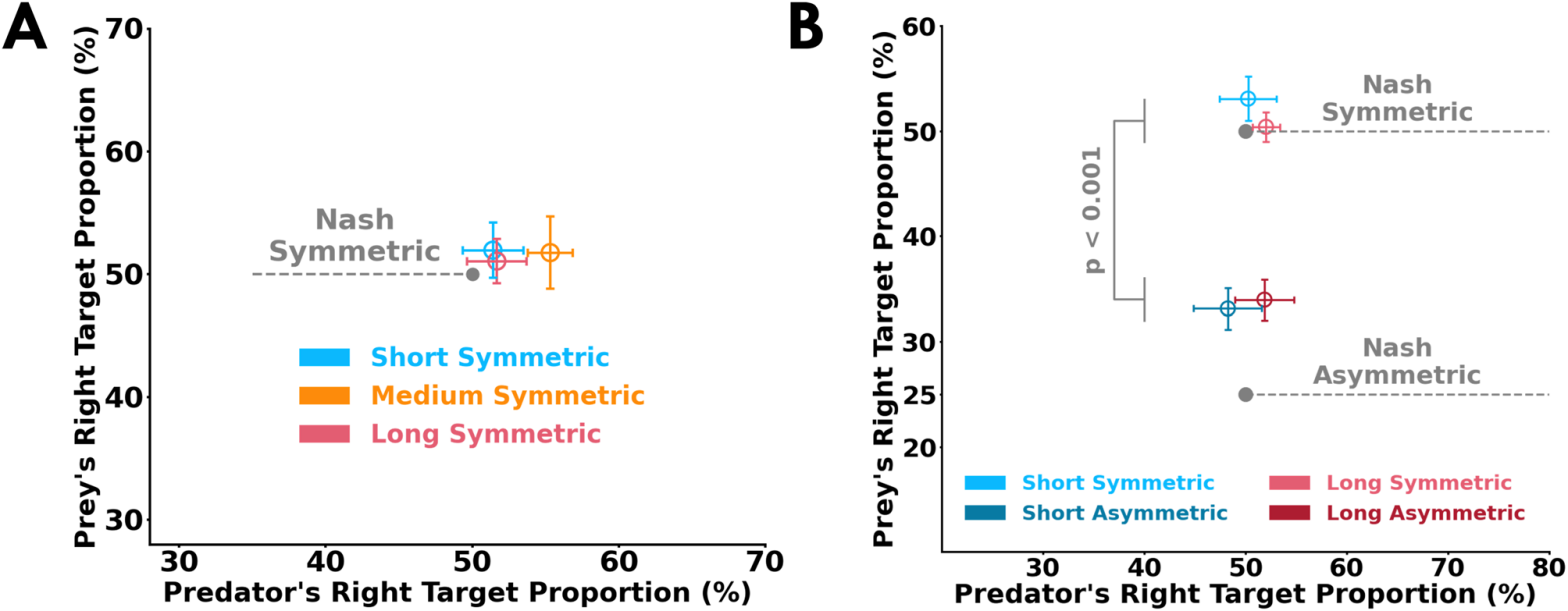
Target selection proportions. (**A**) Average right target proportion (%) of the predator (*x-axis*) and prey (*y-axis*) in Experiment 1. The right target selection proportions of the predator and prey were close to the Nash equilibrium solution for the symmetric reward structure (50% for both predator and prey). (**B**) Average right target proportion (%) of the predator (*x-axis*) and prey (*y-axis*) in Experiment 2. The Nash equilibrium solution (gray circles) for the symmetric (50% for both predator and prey) and asymmetric (50% for predator and 25% for prey) reward structures are shown in (**A**) and (**B**). Error bars represent $\pm \, 1$ standard error. Across both experiments, the predator selected the right target in proportions close to 50%. As expected in the short asymmetric condition, the prey selected the right target in proportions that approached the Nash equilibrium solution. Unexpectedly and against our prediction, the prey target selection approached the Nash equilibrium solution in the long-asymmetric condition—even though they were utilizing sensory evidence of their opponent to select a target (see Fig. 4B,D).

We looked at the proportion that the participants selected the right or left targets. As a reminder, the Nash equilibrium solution for the symmetric reward structure (Fig. 1B) suggests that both the predator and prey should select the two targets in equal proportions. Here, the short-symmetric condition is similar to the cognitive version of the matching pennies game. That is, in the short-symmetric condition participants must decide their target selection prior to moving because they do not have sufficient time to respond to their opponent’s actions. In the medium-symmetric and long-symmetric conditions, the participants could utilize sensory evidence of their opponent to select targets. In Experiment 1, we found no significant effect of the participant’s role (F[1, 22] = 0.24, *p* = 0.62) or trial length (F[2, 44] = 0.87, *p* = 0.42) on right target selection proportions (Fig. 6A). Further, the right target selection proportion did not differ from the Nash equilibrium solution of 0.5 (*p* > 0.99, ${\hat{\theta }}$ < 70.00% for all comparisons), except for the predator in the medium-symmetric condition (*p* = 0.003, ${\hat{\theta }}$ = 91.60%).

For Experiment 2, the Nash equilibrium solution for the asymmetric reward structure (Fig. 1B) suggests that the predator and prey should respectively select the right target in proportions of 50% and 25%. Similar to Experiment 1, we expected the participants to select targets close to the Nash equilibrium solution in the short-asymmetric condition because of its similarity to the cognitive version of the matching pennies game. However, in Experiment 2 we expected that participants would select targets in different proportions depending on the availability of sensory evidence to make decisions. Specifically, we expected that the prey would have a lower proportion of right target selections in the long-asymmetric condition relative to the the short-asymmetric condition. Figure 6B shows the proportion of right target selections for the predator and prey compared to the Nash equilibrium solutions. There was a significant interaction between participant roles and reward condition (F[1, 30] = 19.4, *p* < 0.001). The predator’s right target selection proportions were similar across the reward conditions (*p* = 0.65, ${\hat{\theta }}$ = 53.12%), whereas the prey’s right target selection proportions in the asymmetric conditions were different from the symmetric conditions (*p* < 0.001, ${\hat{\theta }}$ = 93.75%). The predator selected right targets in proportions close to 0.5 in the long-asymmetric condition (*p* > 0.99, ${\hat{\theta }}$ = 68.75%). Unexpectedly, we found that the prey selected the right target in the long-asymmetric condition in similar proportions to the short-asymmetric condition (*p* > 0.99, ${\hat{\theta }}$ = 56.25%). With asymmetries in the reward structure, these results do not support the notion that target selection proportions would differ depending on whether sensory evidence is available for decision-making. It is worthwhile to note that the Nash equilibrium solution analyses do not consider indecisions, which we discuss below.

### Participants had more indecisions with more available time

**Figure 7:**
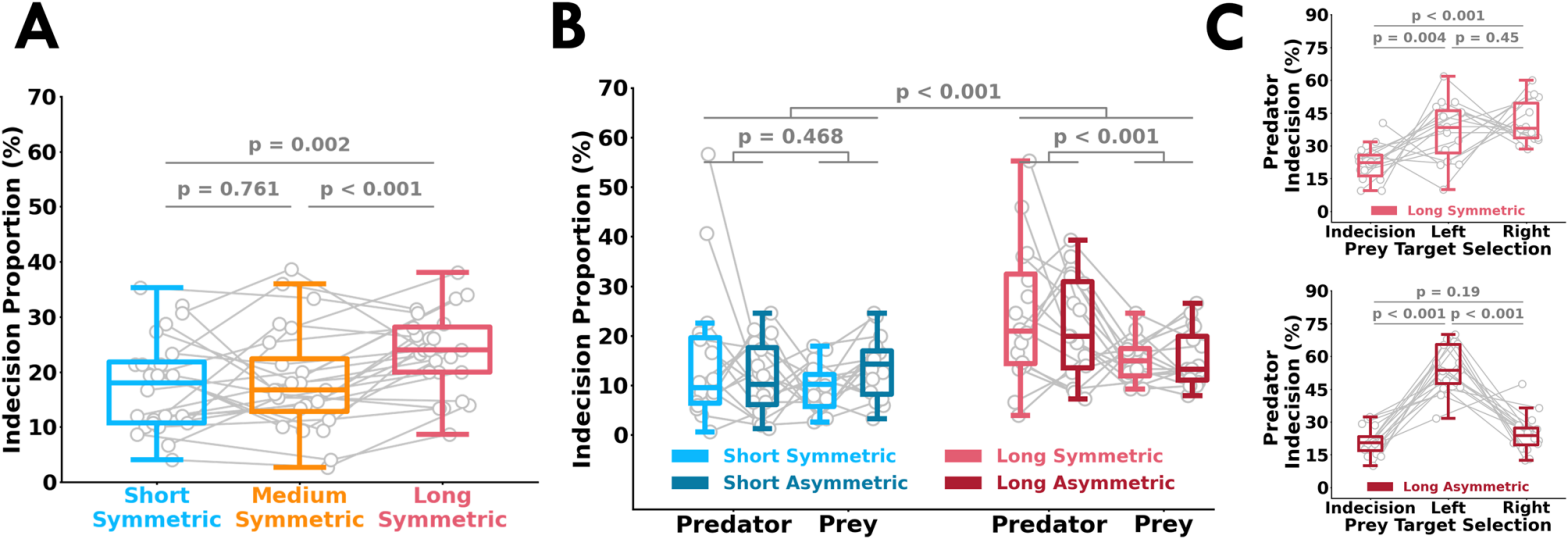
Indecisive behaviour. Proportion of trials (*y-axis*) where participants did not select a target for each condition in (**A**) Experiment 1 and (**B**) Experiment 2. The open gray circles and connecting gray lines correspond to individual participants. Box plots show 25th, 50th, and 75th percentiles. Across both experiments, there were a greater proportion of trials where the participants did not select a target (*y-axis*) in the long conditions relative to the other conditions. Participants did not select a target in the short trial conditions if they did not react in time to the first beep or reach at the necessary speed. Conversely, participants had sufficient time to select a target in the long conditions. More indecisions with more available time may reflect a ‘paralysis-by-analysis’ phenomenon. That is, indecisions may have arisen from participants waiting too long to acquire sensory evidence of their opponent’s intended target selection. Additionally, the predator had higher proportions of indecisions in comparison to the prey in the long conditions. (**C**) Predator indecision proportions (*y-axis*) depending upon the Prey’s target selection or indecision (*x-axis*). In the long-symmetric condition, there was no difference in predator indecision proportions when the prey selected the right or left targets. Conversely, in the long-asymmetric condition the predator had significantly greater indecisions when the prey selected the left target compared to the right target. These results suggest that indecisive behaviour is also influenced by the reward structure.

We examined the proportion of indecision trials where participants did not reach a target. We found a significant effect of trial length on indecisions in Experiment 1 (F[2, 44] = 7.91, *p* = 0.001) and Experiment 2 (F[1, 30] = 53.7, *p* = 0.002). Surprisingly, we found that there were more indecisions in the long-symmetric condition compared to the short-symmetric (*p* < 0.001, ${\hat{\theta }}$ = 79.16%) and medium-symmetric (*p* = 0.001, ${\hat{\theta }}$ = 75.00%) conditions in Experiment 1 (Fig. 7A). This finding was replicated in Experiment 2, where we found that the long conditions had significantly more indecisions relative to the short conditions (*p* <= 0.001, ${\hat{\theta }}$ = 76.56%). Participants did not reach the target in the short conditions if they did not react in time to the first beep or reach at the necessary speed. Conversely, participants had sufficient time to reach a target in the long conditions. More indecisions with more available time may reflect a ‘paralysis-by-analysis’ phenomenon. That is, indecisions may have resulted from participants waiting too long to acquire sensory evidence of their opponent’s intended target selection.

Additionally, in Experiment 2 (Fig. 7B) we found a significant interaction between trial length and participant role (F[1, 30] = 10.94, *p* = 0.002). The predator had significantly more indecisions than the prey in the long conditions (*p* < 0.001,${\hat{\theta }}$ = 68.75%), but not in the short conditions (*p* = 0.46, ${\hat{\theta }}$ = 59.37%). As an exploratory analysis, we tested if the increase in the proportion of predator’s indecisions differed depending upon whether the prey selected the left target, right target, or had an indecision (Fig. 7C). For the long-symmetric condition, the predator’s indecision proportions where the same when the prey selected the left or right target (*p* = 0.45). Conversely, for the long-asymmetric condition we found a greater proportion of predator indecisions when the prey selected the left target in comparison to the right target (*p* < 0.001). That is, the predator was attempting to obtain three points and may have been more focused on the prey moving towards the right target, resulting in more indecisions when the prey selected the left target. These results suggest that indecisive behaviour is influenced by an interplay between sensory evidence and reward structure.

### Decision-making model captured successful behaviour by accumulating sensory evidence

**Figure 8:**
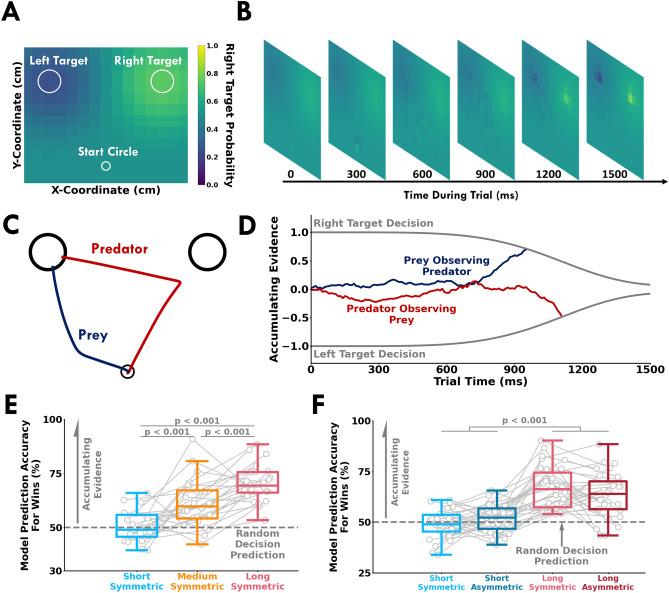
Computational model. The probability of selecting the right target was estimated by discretizing the (**A**) workspace over (**B**) time. Each discretized area was characterized by a beta distribution that sampled the probability an opponent would select the right target ($p_{right}$) given the current location of their cursor. The sampled right target selection probability was used as evidence for the drift-diffusion model. (**C**) Sample hand trajectories of a human pair from a trial in the long-symmetric condition. (**D**) Drift-diffusion model showing accumulation of evidence using the hand trajectories shown in (**C**). The drift-diffusion model accumulates sensory evidence of the opponent’s actions to predict the opponent’s target selection. The model predicts a right or left target selection once the accumulated sensory evidence respectively crosses the upper or lower target decision boundary. A target was selected for the participant based on their role and the predicted target selection for their opponent. For the example shown, the predator observed the prey’s cursor [dark blue trace in (**C**)] and accumulated evidence [dark red trace in (**D**)] that the prey will select the left target (the dark red line crossed the left target decision bound). The predator then correctly selected the left target to win the trial. Conversely, the prey observed the predator’s cursor [dark red trace in (**C**)] and accumulated evidence [dark blue trace in (**D**)] that suggested the predator would go to the right target (the dark blue line crossed the right target decision bound). The prey made a wrong decision based on early accumulated evidence and incorrectly selected the left target in a failed attempt to avoid the predator. Here the drift-diffusion model correctly predicted a left target selection for both the predator and prey. Model prediction accuracy (*y-axis*) for successful decisions in (**E**) Experiment 1 and (**F**) Experiment 2. Model prediction accuracy is the proportion of trials where the predicted decision for a participant matched the observed decision. The open gray circles and connecting gray lines correspond to individual participants. Box plots show 25th, 50th, and 75th percentiles. As expected, the model made random decision predictions ($\approx$ 50%, grey dashed line) in the short trial conditions because there was not enough time to accumulate evidence. Conversely, with more available time to accumulate evidence in the longer trial conditions, the model predicted a participant’s successful behaviour with greater accuracy.

A central idea in our experiment was that a participant observed their opponent’s cursor to accumulate sensory evidence and predict their target selection. Upon predicting an opponent’s intended target selection, a participant could then select a target appropriate to their role. We tested whether a drift-diffusion model could capture the decision-making behaviour of the participants. Sensory evidence was calculated as the probability that the opponent would select the right (or left) target given the current position of the opponent’s cursor (Fig. 8A,B). In this context, the drift-diffusion model accumulates this sensory evidence to predict a right or left target selection for the opponent (Fig. 8C,D), and a target was selected based on the participants assigned role. For each participant and experimental condition, we calculated the proportion of trials where the model’s predicted target selection matched the actual target selection. Here, we focus on *model prediction accuracy* for winning trials since we were primarily interested in how utilizing sensory evidence promoted successful decision-making behaviour. We also report the overall *model prediction accuracy* for wins, losses, and indecisions (see Supplementary B). There was a significant main effect of trial length on the *model prediction accuracy* for successful behaviour in Experiment 1 (F[2, 44] = 37.91, *p* < 0.001) and Experiment 2 (F[1, 30] = 61.36, *p* < 0.001). As expected for the short trial conditions, the model predicted at the chance level because there was insufficient time to accumulate sensory evidence of the opponent’s intended target selection. In Experiment 1 and 2 (Fig. 8E,F), the *model prediction accuracy* was significantly higher with more available time (*p* < 0.001, ${\hat{\theta }}$ > 75.00% for all comparisons). Thus, with more available time, the model was able to accumulate sensory evidence of the opponent’s action to appropriately predict their decision. Our model aligns with the idea that humans accumulate sensory evidence to correctly predict the action intention of their opponent.

Taken together, our results suggest that participants who exploited time and relied on sensory evidence of their opponent to make decisions were more successful. The drift-diffusion model was able to predict successful behaviour by accumulating sensory evidence of an opponent’s actions. Interestingly, with more available time participants often failed to reach a target. This finding may arise from a paralysis-by-analysis phenomenon, where waiting too long to acquire sensory evidence of an opponent’s intended action was detrimental to performance.

## Discussion

During a competitive sensorimotor task, our findings suggest that humans exploit available time to accumulate sensory evidence of their opponent’s actions to make decisions. With more available time participants increasingly relied on sensory evidence of their opponent’s movements, which is supported by the observed tracking and avoidance behaviour. We also questioned whether sensory information of an opponent’s actions would cause participants to shift away from selecting targets in proportions that approach an optimal game theoretic prediction (Nash equilibrium solution). Participants selected targets in proportions that approached the Nash equilibrium solution, similar to cognitive decision-making tasks^10^, despite utilizing online sensory evidence of their opponent. Surprisingly, we also found that participants were less likely to select a target with more available time. This finding may reflect a paralysis-by-analysis phenomenon. That is, participants waited too long to accumulate sensory evidence of their opponent’s intended target selection, which resulted in indecisive behaviour. We also captured online and interactive decision-making behaviour using a drift-diffusion model.

The different time conditions in our experiment allowed us to manipulate the amount of sensory evidence used to make a decision. We found that participants exploited more available time with an increase in trial length. Specifically we found with more available time that participants delayed the time of last change in movement direction and displayed a greater $t_{0.75}$ time, both of which correlated with successful performance. Participants may have exploited time for two reasons. First, participants may have used this additional time to accumulate sensory evidence of their opponent’s actions. Second, participants could also have been simultaneously attempting to keep their eventual target selection unpredictable for a longer portion of the trial. Our findings suggest that humans exploit available time to remain unpredictable and to accumulate sensory evidence of other’s actions during competitive interactions.

The predator and prey could win a trial by using sensory evidence to respectively track and avoid each other. We captured tracking or avoidance behaviour of the participants by considering the relative position of both participants. The *mutual location probability* remained close to 0.5 throughout the short condition trials, and early on during the medium and long conditions. In the short conditions, participants could not react to the sensory evidence of their opponent’s actions because they did not have sufficient time. Further, as discussed earlier, the early sensory evidence during the medium and long conditions was weakly indicative of a participant’s eventual target selection. Thus, it would not be useful for participants to react to their opponent’s actions early on in the medium and long condition trials. However, as a trial progressed, the *mutual location probability* showed tracking and avoidance behaviour in the medium and long condition trials. The idea that humans increasingly rely on sensory information as a trial progresses aligns with past research that showed humans delay their decision onset when there is noisy sensory evidence during initial stimulus presentation^32^. During collaborative tasks, utilizing early sensory evidence of a partner is important for achieving a common goal^17,18,33–35^. Observation of other’s actions not only provides an understanding of their current goals but aids in the prediction of future actions^30^. In our study, we found participants reacted to their opponent’s actions that were predictive of target selection.

With more available time the participants used a broader set of movement strategies. Participants could have used more complex trajectories to react to their opponent, remain unpredictable, or even mislead their opponent about their intended target selection. As discussed in the paragraph above, there was clear evidence that participants were reacting to their opponent’s actions since they displayed tracking and avoidance behaviour. Additionally, movements may have been in part used by a participant to mislead or deceive their opponent about their eventual target selection. Such deceptive movements are commonly observed in interactive sports^36,37^. In the current study, it is unclear to what extent the movements were used to mislead the opponent versus react to the opponent’s movements. It would be interesting to study the effect of individual differences on movement strategies and the relation to task performance. Further, movement strategies may be influenced by physiological factors like age and gender, experiential factors like prior gaming experience and sports experience, and socioeconomic factors.

Iterative decision-making during cognitive-based tasks has been studied and characterized extensively from a game theoretic perspective^5,7,9^. With the symmetric reward structure, we found that the participants selected targets in equal proportions that aligned with the Nash equilibrium solution. We then used an asymmetric reward structure to further examine decision-making behaviour when participants have sensory evidence of one another. During cognitive decision-making tasks, past research using an asymmetric reward structure in the matching pennies game have reported that humans^10^, chimpanzees^10^, and pigeons^38^ select decisions that approached the Nash equilibrium solution. The short conditions used in our experiment were analogous to cognitive decision-making tasks since participants did not have sufficient time to utilize sensory evidence of their opponent. Expectedly, we found during short conditions that participants proportionally selected targets in a manner that approached the Nash equilibrium solution. However, it is possible that sensory evidence of an opponent prior to making a decision might shift decision-making behaviour away from the Nash equilibrium solution. Interestingly, we observed that the predator and prey also proportionally selected targets in the long conditions in a manner that approached the Nash equilibrium solution—despite clearly utilizing sensory evidence to perform tracking and avoidance behaviour. One possibility is that participants relied on sensory evidence when their opponents selected a target early, but then resorted to randomly selecting targets when they had insufficient sensory evidence of their opponents intended actions. A worthwhile future direction is to further examine the interplay of online sensory evidence and optimal decision-making from a game theoretic perspective.

Our study bridges research between perceptual decision-making and human sensorimotor interactions. The neuroeconomics and psychology literature have studied human-human decision-making by adopting classical cognitive games^5,7,9^. Perceptual decision-making studies with individual rodents^23^, non-human primates^24,25^ and humans^26,27^ have established a strong link between sensory evidence and neural activity in premotor and prefrontal cortices. Past work studying collaborative human-human sensorimotor interactions have addressed communication of intentions^16,34,35,39^, skill level^40,41^, interaction forces^18,20^ and role specialization^17,42^. Researchers have used differential game theory to model continuous sensorimotor control during physical human interactions^19,43–46^. However, these interaction scenarios differ from perceptual decision-making, where each interacting individual utilizes sensory evidence to make a decision and select an action. Braun and colleagues used continuous sensorimotor versions of classical cognitive games to study haptic interactions between humans and between trial decision strategies^12,47^. The focus of these studies was to characterize *between trial* decision-making strategies of the participants, but not the influence of online sensory evidence on *within trial* decision-making. To our knowledge, we are the first to characterize the influence of online sensory evidence on perceptual decision-making during human-human interactions.

Successful decision-making during interactions with other humans requires the ability to predict the outcomes of another person’s actions^30^. Studies have reported neural activity in premotor areas of the human brain when observing^48^ or predicting^49,50^ others’ actions. Previously, models based on game theory that represent the goals and actions of other interacting humans have been implemented for cognitive decision-making tasks^51^ and continuous interaction tasks^19,45^. Behaviorally, participants most likely won by chance in the short condition trials because they did not have sufficient time to accumulate sensory evidence. Accordingly, the model predicted winning decisions close to the chance level. However, a participant most likely won in the longer trials by observing their opponent’s cursor and correctly predicting their target selection. Accordingly, our model predicted a participant’s winning decisions with greater accuracy when there was more time available to accumulate sensory evidence.

Our model was motivated by the idea that participants utililze sensory evidence of their opponents actions in an attempt to predict their eventual target selection. There are several different proposed models that have been used to capture perceptual decision-making: (1) a perfect accumulator model, such as the drift-diffusion model used in this study^28^, (2) leaky accumulator models^52^, or urgency-gating models^53^. Functionally, the drift-diffusion model accumulates evidence throughout the trial, whereas the urgency-gating models rely more heavily on sampled evidence later in time. In this paper, we focused on a drift-diffusion model that has been used extensively to capture decision-making behaviour during perceptual tasks by accumulating sensory evidence^21,29,54^. Overall, our model did well to capture decision-making behaviour of the participants (see Fig. 8, Supplementary B). Additionally, we tested a leaky accumulator and urgency-gating model (Supplementary C). All three models did well to capture the data, with the perfect accumulation drift-diffusion model using the least number of free parameters (Fig. 8). More recently it has been suggested that both evidence accumulation and urgency-gating are used to make decisions^55,56^. To what extent the nervous system relies on evidence accumulation, urgency-gating, or both remains an open question. It could be interesting to compare the performance of different decision-making models in predicting “change of minds”, where participants make a change in decision and movement just prior to selecting a target^57^.

Surprisingly, in both experiments we found a greater proportion of indecisions in the long trials—despite participants having ample time to physically reach the target. In the short condition, participants would not reach a target if they had a poor reaction time, slow movement, or attempted to select a target based on observing their opponent. However, participants had sufficient time to reach a target in the long condition trials, but had significantly more indecisions. More indecisions with more available time may reflect a paralysis-by-analysis phenomenon. That is, late decisions may have resulted from participants waiting too long to acquire and act upon sensory evidence of their opponent’s intended action. Paralysis-by-analysis has been observed in decision-making scenarios across various types of social interactions^58^ including sport^59^ and economics^60,61^. Late decisions may have resulted from participants waiting too long to acquire and act upon sensory evidence of their opponent’s intended action.

We also observed that the predator had more indecisions in comparison to the prey in the long conditions. Exploring this result, in the symmetric condition there were no differences in predator indecision when the prey selected the right or left target. Yet in the long-asymmetric condition we found that predator indecision proportions were greater when the prey selected the left target when compared to the right target in the long-asymmetric condition, but not in the long-symmetric condition. For the asymmetric reward condition, the predator received three points for selecting the right target when the prey also selected the right target. The predator was attempting to obtain three points and may have been more focused on the prey selecting the right target, resulting in more indecisions when the prey selected the left target. That is, the predator may have been waiting longer to accumulate sensory evidence on whether the prey would select the right target, which lead to a greater proportion of indecisions. Asymmetries in the reward structure seems to influence the tendency to wait for more sensory evidence of another person’s actions when it is linked to greater reward (or loss), which may come at the cost of more indecisions.

A limitation of our model is that it did not predict indecisions with good accuracy. Indecisions can result from delayed prediction of the opponent’s intended target selection and movement requirements to select a target within the available time. While our model accounted for the delays of sensing and predicting the opponent’s intended target selection, it did not incorporate delays associated with physically reaching a desired target. Nevertheless, the model was generally effective in predicting overall target selection across all trials (Fig. 8, Supplementary B). There may be an optimal time to make interactive decisions that balances acquiring sufficient sensory evidence while satisfying time constraints. An interesting direction for future research is understanding the tradeoff between using online sensory evidence and indecisions, as well as developing models that better predict indecisive behaviour.

How humans make decisions while interacting with other humans is highly relevant in everyday social life, sport, machine learning (i.e., multiple ‘agents’), and economics. Our work advances our understanding of how two interacting humans jointly make decisions and select actions. Across two experiments, we tested the influence of available time and reward structure on interactive, decision-making behaviour. We found that humans exploit available time to gather sufficient evidence about the action intention of others, and use this information to generate an appropriate motor response. We were able to capture decision-making behaviour of competing humans using a drift-diffusion model. Insights on how humans utilize sensory evidence to predict the action intentions of others may enable more seamless human-machine interfaces, which has important applications for augmented reality and robot-guided rehabilitation. Our ability to observe others and make online decisions is highly relevant across many domains, spanning sociology, sport, interactive technology, and economics.

## Methods

### Participants

Twenty-four individuals (12 human pairs, 13 female, age 18–30 years) participated in Experiment 1, and 32 individuals (16 human pairs, 17 female, age 18–30 years) participated in Experiment 2. All participants provided informed consent to participate in the experiment and the procedures were approved by the University of Delaware’s institutional review board. All methods were performed in accordance with the relevant guidelines and regulations.

### Apparatus

For both experiments, we used two endpoint KINARM robots (Fig. 1A; BKIN Technologies, Kingston, ON) that are able to interact with each other in real-time. Each participant of a human pair was seated on an adjustable chair in front of one of the endpoint robots. Each participant grasped the handle of a robotic arm and made arm movements in the horizontal plane. A semi-silvered mirror blocked vision of the upper limb and displayed virtual images (e.g., targets, cursors) from an LCD screen. Hand position was recorded at 1000 Hz and stored offline for data analysis.

### Protocol

#### Experimental design for Experiment 1 and Experiment 2

We designed a competitive motor task based on the matching pennies game. In our task, each participant controlled a circular cursor (1 cm diameter) located at the position of the robot handle and hand. The participants saw their cursor and their opponent’s cursor projected on their respective screens during the trial. Each trial began once both participants were in their respective start position (1.5 cm diameter). After a short, randomized delay (250–1000 ms), participants heard a first beep and two targets appeared. The left and right targets (4 cm diameter rings) were separated by 20 cm and were 15 cm forward relative to the start position (Fig. 1A). Participants were instructed to move their cursor into a target of their choice before they heard a second beep that indicated the end of the trial. Once a participant entered a target, their cursor remained there until the second beep.

For each human pair, participants were randomly assigned as the ‘predator’ or the ‘prey’ before the start of the experiment. The predator won the trial by selecting the same target as the prey (e.g., both predator and prey selected the right target). Conversely, the prey won the trial by selecting the opposite target of the predator (e.g., prey selected the right target but the predator selected the left target). Additionally, a participant won the trial if they selected a target and their opponent did not select a target prior to the second beep. Both participants lost the trial if they failed to reach a target before the second beep. In addition to a base compensation of $5.00 USD, we informed the participants that they would receive a performance-based compensation up to $5.00 USD based on the points that they won. Participants received the full $10.00 USD once they completed the experiment, irrespective of their performance.

#### Experiment 1 design

The goal of Experiment 1 was to determine how humans utilize sensory evidence of their opponents actions when making decisions. Participants received 1 point for winning a trial and 0 points for losing a trial according to the symmetric reward structure for the matching pennies game (Fig. 1B). To manipulate the ability to accumulate sensory evidence, human pairs performed three conditions with differing available time: (1) short-symmetric (500 ms), (2) medium-symmetric (850 ms), and (3) long-symmetric (1500 ms). The short-symmetric condition did not provide sufficient time to observe an opponent while selecting a target. The medium-symmetric and long-symmetric conditions increasingly afforded a participant the ability to observe their opponent’s cursor when making a decision to select a target. We informed the participants about the available time at the start of each condition. Human pairs completed 150 trials per condition. Condition order was counterbalanced.

#### Experiment 2 design

The goal of Experiment 2 was to further test the idea that humans utilize sensory evidence of their opponents actions when making decisions. We also tested a secondary hypothesis that game theoretic predictions for target selection would not be preserved when the players have available time to utilize sensory evidence of their opponent’s actions. The asymmetric reward condition was similar to the symmetric reward condition, with the exception that the predator received 3 points if both the predator and prey selected the right target (Fig. 1B). We manipulated both available time and reward structure using the four conditions: (1) short-symmetric, (2) long-symmetric, (3) short-asymmetric, and (4) long-asymmetric. The symmetric reward structure was the same as that of Experiment 1 (Fig. 1B). We informed the participants about the available time and the reward condition at the start of each condition. Human pairs each completed 150 trials of the 4 condition combinations. We counterbalanced the order of the reward conditions and available time.

### Data analysis

#### Principal component analysis

For each trial where the participant reached a target, we sampled the x-coordinate of the cursor trajectory at 100 equidistant time points. Here, we sampled the trajectory from the time the cursor left the start circle to the time when the cursor entered the target. Using the x-coordinate data from all trials within a condition, we used principal component analysis to calculate the percentage of variance explained by each orthogonal principal component.

#### Movement time

For each participant and trial, we measured the time from the start of the trial (first beep) till they reached the target (ms).

#### Time of last change in movement direction

We discretized the cursor trajectory into segments of length 0.5 cm. We segmented the trajectory from the start of the trial to the time when the cursor entered the target. We calculated the direction of each segment in cartesian space. We searched backward in time until a segment was found that was 15 degrees different from the last segment. The moment in time of the initial point of this segment was considered as the *time of last change in movement direction* before reaching the target.

#### Exploiting available time: target choice probability

Here we were interested in quantifying whether participants exploited available time. We calculated *target choice probability* (Eq. 1), which is the probability that a participant selects the right or the left target given that the cursor position is respectively on the right side or the left side of the workspace. Specifically,

1 $$\begin{aligned}&Target \; Choice \; Probability = \nonumber \\&p(cursor_{right} | \; target_{right}) \cdot p(target_{right}) + p(cursor_{left} | \; target_{left}) \cdot p(target_{left}) \end{aligned}$$

We computed the *target choice probability* at equidistant time samples in the trial. We then normalized the time from the start to the end of the trial. We used trials where a participant reached a target. For each participant, we determined the moment in normalized time at when the *target choice probability* crossed 75% ($t_{0.75}$). A greater $t_{0.75}$ time suggests participants are exploiting available time before selecting a target.

#### Tracking and avoidance behaviour of the predator and prey: mutual location probability

Here we were interested in whether participants positioned their cursor relative to their opponent’s cursor, which can provide insight into tracking and avoidance behaviour. Accordingly, we calculated the probability that the cursor positions of the predator and the prey are on the same side of the workspace (Eq. 2):

2 $$\begin{aligned}&Mutual \; Location \; Probability = \nonumber \\&p(prey_{right} | \; predator_{right}) \cdot p(predator_{right}) + p(prey_{left} | \; predator_{left}) \cdot p(predator_{left}) \end{aligned}$$

A participant’s *mutual location probability* is not defined after the participant selects a target because the participant’s cursor remained fixed at the selected target. Consequentially, the mutual location probability was different for the predator and prey since they most likely reached a target at different times during the trial. Thus, we normalized time from the start of a trial to when the participant entered a target. We calculated the *mutual location probability* at equidistant samples in the trial. A *mutual location probability* greater than 0.5 suggests tracking behaviour by the participant. Conversely, a *mutual location probability* less than 0.5 suggests avoidance behaviour.

#### Behaviour related to successful performance

We correlated *target choice probability* time $t_{0.75}$ differences of the predator and prey to win proportions. We correlated the *time of last change in movement direction* differences of the predator and prey to the win proportions. Pearson’s correlation was performed for each condition.

#### Target selection proportions and game theoretic predictions

We analyzed the target selection proportions of the participants. Here we considered trials where both participants reached a target. We calculated the proportion of trials where the predator and prey selected the right target. We calculated the Nash equilibrium solution for the symmetric and the asymmetric reward structures of the matching pennies game. The Nash equilibrium solution corresponds to randomly selecting the right or left targets in a proportion, such that each participant has nothing to gain by deviating from that particular strategy.

To calculate the Nash equilibrium solution, we assume that predator and prey select the right target in proportions $P_{predator}$ and $P_{prey}$, respectively, in accordance with the Nash equilibrium solution. We also assume a general form of the reward structure (Fig. 1B) as follows:

$$\begin{aligned} \begin{pmatrix} a_{11}, b_{11} &{}\quad a_{12}, b_{12} \\ a_{21}, b_{21} &{}\quad a_{22}, b_{2, 2} \end{pmatrix} \end{aligned}$$

The corresponding values of the rewards for the symmetric reward structure are $a_{11}=a_{22}=b_{12}=b_{21}=1;\; a_{12}=a_{21}=b_{11}=b_{22}=0$, and the asymmetric reward structure are $a_{11}=3;\; a_{22}=b_{12}=b_{21}=1;\; a_{12}=a_{21}=b_{11}=b_{22}=0$. Equating the expected reward for the right and left target selection of the predator, we obtain

3 $$\begin{aligned} P_{prey} \times a_{11} + (1 - P_{prey}) \times a_{12} = P_{prey} \times a_{21} + (1 - P_{prey}) \times a_{22} \end{aligned}$$

Similarly, equating the expected reward for the right and left target selection of the prey yields

4 $$\begin{aligned} P_{predator} \times b_{11} + (1 - P_{predator}) \times b_{21} = P_{predator} \times b_{12} + (1 - P_{predator}) \times b_{22} \end{aligned}$$

We substituted reward values for the symmetric reward structure and asymmetric reward structure into (Eq. 3) and (Eq. 4) to obtain the corresponding Nash equilibrium solutions. For the symmetric reward structure, the Nash equilibrium solution specifies that the prey and predator should respectively select the right target in proportions of 50% ($P_{prey} = 0.5$) and 50% ($P_{predator} = 0.5$). For the asymmetric reward structure, the Nash equilibrium solution specifies that the prey and predator should respectively select the right target in proportions of 25% ($P_{prey} = 0.25$) and 50% ($P_{predator} = 0.5$).

#### Proportion of targets not selected

We calculated the proportion of trials where a participant did not reach a target, indicating indecisive behaviour. Here, we were interested in whether sensory evidence of the opponent’s cursor would alter the percentage of indecisions.

### Decision-making model

We developed a computational model to explain a participant’s decision-making process. We posited that each participant observes the opponent’s cursor and accumulates this evidence to predict their opponent’s target selection. To capture this process, we used a drift-diffusion model^28,29,62^.

We adopted a probabilistic approach to compute the evidence based on the opponent’s cursor position. The two-dimensional reaching workspace was discretized into smaller spatial areas (Fig. 8A). Collectively, the discretization of the workspace represented a probability map. Probability maps were maintained at every 10 ms of time (Fig. 8B). The probability maps were initialized at the beginning of an experimental condition. Discretized areas close to the right target represented higher right target selection probabilities, whereas the discretized areas close to the left target represented lower right target selection probabilities.

Each discretized area was represented by a beta distribution with hyperparameters $\alpha$ and $\beta$. We updated the beta distributions after each trial based on the observed total number of trials where a target was selected (*n*) and the number of trials where the right target was selected (*k*). From Bayes theorem (Eq. 7), the posterior distribution [$\text {p}(p_{right} | \; k, n, \alpha _{prior}, \beta _{prior})$] is given by the product of the likelihood [${\mathcal {L}}(p_{right}|\; k, n)$] and the prior distribution [$p(p_{right}|\; \alpha _{prior}, \beta _{prior})$] as follows:

5 $$\begin{aligned} p(p_{right} | \; k, n, \alpha _{prior}, \beta _{prior}) \propto {\mathcal {L}}(p_{right}|\;k, n ) \; p(p_{right}|\; \alpha _{prior}, \beta _{prior}) \end{aligned}$$

The prior beta distribution (Eq. 8) is

6 $$\begin{aligned} p(p_{right}|\; \alpha _{prior}, \beta _{prior})&= \frac{1}{B(\alpha _{prior}, \beta _{prior})} \; p_{right} \, ^ {\alpha _{prior} - 1} \; (1 - p_{right}) ^ {\beta _{prior} - 1} \end{aligned}$$

where *B* represents the Beta function. The likelihood function (Eq. 9) is a binomial distribution of the following form

7 $$\begin{aligned} {\mathcal {L}}(p_{right}|\;k, n ) = \frac{(n)!}{k!(n - k)!} \; p_{right}\,^{k} \; (1 - p_{right})^{n - k} \end{aligned}$$

As a reminder, *n* is the total number of trials and *k* is the number of trials that a participant selected the right target. Given conjugate priors, the posterior distribution is a beta distribution (Eq. 10) with hyperparameters (Eqs. 11 and 12) according to

8 $$\begin{aligned} p(p_{right}|\; k, n, \alpha _{prior}, \beta _{prior})&= \frac{1}{B(\alpha _{post}, \beta _{post})} \; p_{right} \, ^ {\alpha _{post} - 1} \; (1 - p_{right}) ^ {\beta _{post} - 1} \end{aligned}$$

9 $$\begin{aligned} \alpha _{post}&= k + \alpha _{prior} \end{aligned}$$

10 $$\begin{aligned} \beta _{post}&= n - k + \beta _{prior} \end{aligned}$$

We sampled the probability that the opponent selects the right target using the posterior beta distribution (Eq. 10). The sensory evidence based on the opponent’s cursor position (*E*) was calculated using the right target probability ($p_{right}$) according to

11 $$\begin{aligned} E = ln\left( \frac{p_{right}}{1 - p_{right}}\right) \end{aligned}$$

A drift-diffusion model was used to accumulate evidence ($E_{acc}$) and predict target selections for the opponent (Fig. 8D). The evidence was accumulated every 10 ms during the trial as follows:

12 $$\begin{aligned} dE_{acc} = \mu Edt + \varepsilon \end{aligned}$$

Thus, $E_{acc}$ represents the accumulating sensory evidence. The parameter $\mu$ represents the gain on the drift rate. We obtained the best value of $\mu$ as 0.007 that maximized model prediction accuracy. Gaussian noise ($\varepsilon$) was randomly sampled with a zero mean and standard deviation ($\sigma$) of 0.02. A delay of 200 ms was incorporated into the accumulation process to accommodate sensory processing delays^57,63^. The evidence was accumulated until it reached the top boundary or the bottom boundary to respectively predict that the opponent selects the right or left target. These target selection boundaries were modelled with the sigmoidal function,

13 $$\begin{aligned} b(t) = 1 - 0.5 (1 - e ^ {-\frac{5 \cdot t}{\gamma }}) \end{aligned}$$

Here, $\gamma$ was used to control the time when the boundaries collapse. For each condition, $\gamma$ was selected such that $b(t) = 0.5$ was aligned with the median *time of last change in movement direction*. A target was selected for the participant based on their role and the predicted target for the opponent.

#### Model prediction accuracy

We calculated the proportion of trials where the model predicted target selection matched the observed target selection. We focus primarily on the model prediction accuracy for wins to capture successful decision-making behaviour of participants, but also report model prediction accuracy when considering wins, losses, and indecisions (see Supplementary B). To obtain the drift rate parameter ($\mu = 0.007$) for the drift-diffusion process, we used a brute force grid search method that maximized the overall *model prediction accuracy*. Here, the loss function was the negative of the mean model prediction accuracy across all the participants. We obtained a single drift rate parameter across conditions to avoid overfitting the data.

### Statistical analysis

We used analysis of variance (ANOVA) as omnibus tests to determine whether there were main effects and interactions. In Experiment 1, we used a 3 (available time: short, medium, long) $\times$ 2 (role: predator, prey) mixed ANOVA to test for main effects and interactions separately for each dependent variable. For Experiment 2, we used 2 (available time: short, long) $\times$ 2 (reward condition: symmetric, asymmetric) $\times$ 2 (role: predator, prey) mixed ANOVA to test for main effects and interactions separately for each dependent variable. We followed up the omnibus tests with mean comparisons using nonparametric bootstrap hypothesis tests (n = 1,000,000)^64–66^. The Holm-Bonferroni procedure was used to account for multiple comparisons. We computed the common language effect sizes (${\hat{\theta }}$) for all mean comparison^67^. Significance threshold was set at $\alpha$ = 0.05.

## Supplementary Information

Supplementary Information.

## Data Availability

The datasets generated and analyzed during the current study are available on the Open Science Framework: https://osf.io/bgf7t/.
