# Supplementary material for: Humans utilize sensory evidence of others’ intended action to make online decisions
Source: Sci Rep. 2022 May 25;12:8806. doi: 10.1038/s41598-022-12662-y (PMC9132989; doi:10.1038/s41598-022-12662-y)
Supplement: Supplementary file 1 — Supplementary Information. [file 41598_2022_12662_MOESM1_ESM.pdf]

Supplementary A

Tracking and avoidance behaviour: correlations and time lags between predatory and prey movement

The predator and prey respectively exhibited tracking and avoidance behaviour (**Fig. 4B,4D**). Here we also examine the time lagged relationship between the predator and prey's movements by correlating their lateral hand velocities. We segmented a participant's lateral hand velocity by sliding a window of 100 ms in steps of 10 ms from the start to end of the trial. For any given segment, we correlated it with lagged 100 ms segments of the opponent's lateral hand velocity, using lags in steps of 10 ms and maximum lag of 500 ms. We identified the maximal correlation and the corresponding lag. We repeated the procedure for each segment to obtain a vector of correlations and lags along normalized time. We normalized the correlations to the absolute maximum of the correlations within the trial. Here, we define the maximal correlation as *movement correlation* and the corresponding lags as *movement correlation lag*.

We plotted the movement correlation and movement correlation lag along normalized time for Experiment 1 (**Fig. S1A & S1C**, respectively) and Experiment 2 (**Fig. S1B & S1D**, respectively). A *movement correlation* greater than zero with high *movement correlation lags* corresponds to tracking behaviour by a participant. Conversely, a *movement correlation* less than zero with a high movement correlation lag corresponds to avoidance behaviour. A *movement correlation* and *movement correlation lag* close to zero represents random relative movements.

To assess the tracking and avoidance behaviour we looked at the average *movement correlation* and *movement correlation lags* along normalized time for Experiment 1 (**Fig. S1E & Fig. S1G**, respectively) and Experiment 2 (**Fig. S1F & Fig. S1H**, respectively). For *movement correlation*, we found a significant interaction between available time and participant role (predator or prey) in Experiment 1 ($F[2, 44] = 14.95$, $p < 0.001$) and Experiment 2 ($F[1, 30] = 42.07$, $p < 0.001$). In both experiments, the predator displayed significantly greater tracking behaviour in the long conditions compared to short conditions ($p < 0.005$, $\hat{\theta} > 71\%$, for all comparisons). Conversely, the prey displayed significantly greater avoidance behaviour in the long conditions compared to short conditions ($p < 0.001$, $\hat{\theta} > 75\%$, for all comparisons). For *movement correlation lag*, we found a significant effect of available time in Experiment 1 ($F[2, 44] = 867.58$,

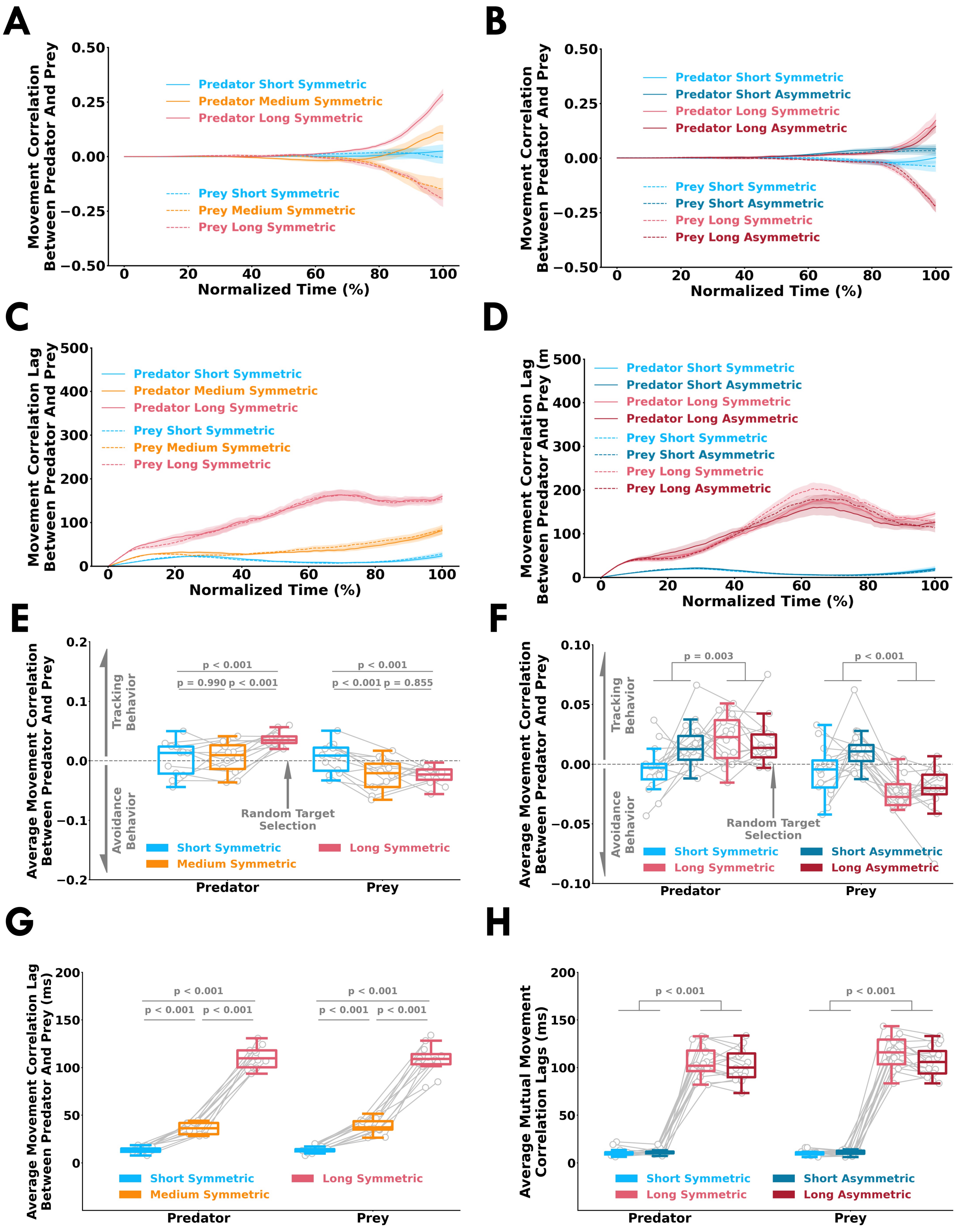

Figure S1: Movement Correlation Between Human Pairs. Maximum correlation between a participant's current movement and their opponent's movement is termed as *movement correlation*. Corresponding time lag (milliseconds) where a maximum correlation was observed is termed as *movement correlation lag*. *Movement correlation* (*y-axis*) across normalized time (*x-axis*) for **(A)** Experiment 1 and **(B)** Experiment 2. Error ribbons represent ± 1 stan-

dard error. *Movement correlation lag* (*y-axis*) across normalized time (*x-axis*) for **(C)** Experiment 1 and **(D)** Experiment 2 .*Movement correlation* (*y-axis*) averaged across normalized time for **(E)** Experiment 1 and **(F)** Experiment 2. *Movement correlation lag* (*y-axis*) averaged across normalized time for **(G)** Experiment 1 and **(H)** Experiment 2. The open gray circles and connecting gray lines correspond to individual participants. Box plots show 25th, 50th, and 75th percentiles. Complementing the mutual location probability (Fig. 4B,4D), these results suggest that the participants were observing their opponents and reacting to their movements.

$p < 0.001$) and Experiment 2 ($F[1, 30] = 1210.37$, $p < 0.001$). In both experiments, participants had lagged movement relative to their opponent in the long conditions compared to short conditions ($p < 0.001$, $\hat{\theta} = 100\%$, for all comparisons). The delayed correlations in the long conditions suggest that the participants were observing and reacting to their opponent's movements.

Supplementary B

Model prediction accuracy considering wins, losses and indecisions

We quantified the performance of the drift-diffusion model by calculating the *model prediction accuracy*. Here, the *model prediction accuracy* is the proportion of trials where the drift-diffusion model predicted target selection matched the observed target selection. We focused on the drift-diffusion model prediction accuracy for the winning trials (**Fig. 8E, 8F**) in the Results section because we were primarily interested in successful decision-making behaviour that relied on accumulating sensory evidence of an opponent. Nevertheless, here we report the *model prediction accuracy* relative to chance prediction levels when considering wins, losses and indecisions for Experiment 1 (**Fig. S2A**) and Experiment 2 (**Fig. S2B**). To calculate the chance prediction levels for each condition, we used a resampling method that pseudorandomly sampled target selections (left, right or indecision) according to the observed target selection frequency. We calculated the proportion of matches between the pseudorandomly sampled target selections and the observed target selections. We repeated the simulation 100,000 times and averaged the proportion of matches to obtain the chance prediction level. We then subtracted the chance prediction level from the model prediction accuracy based on the drift-diffusion model, such that 0% corresponds to the chance prediction level.

There was a significant main effect of available time on *model prediction accuracy* relative to chance in Experiment 1 ($F[2, 44] = 11.56$, $p < 0.001$) and Experiment 2 ($F[1, 30] = 52.42$, $p < 0.001$). The model prediction accuracy relative to chance was significantly higher with more available time ($p < 0.05$, $\hat{\theta} > 71\%$ for all comparisons). Additionally, across both experiments and conditions, the *model prediction accuracy* relative to the chance level was significantly greater than zero ($p < 0.05$, $\hat{\theta} > 66\%$ for all comparisons). The model prediction accuracies are significantly greater than chance for the short conditions because the Bayesian updates are able to capture biases in target selection. As expected, the *model prediction accuracy* was greatest in the long conditions because the drift-diffusion model utilized accumulated sensory evidence to make decisions.

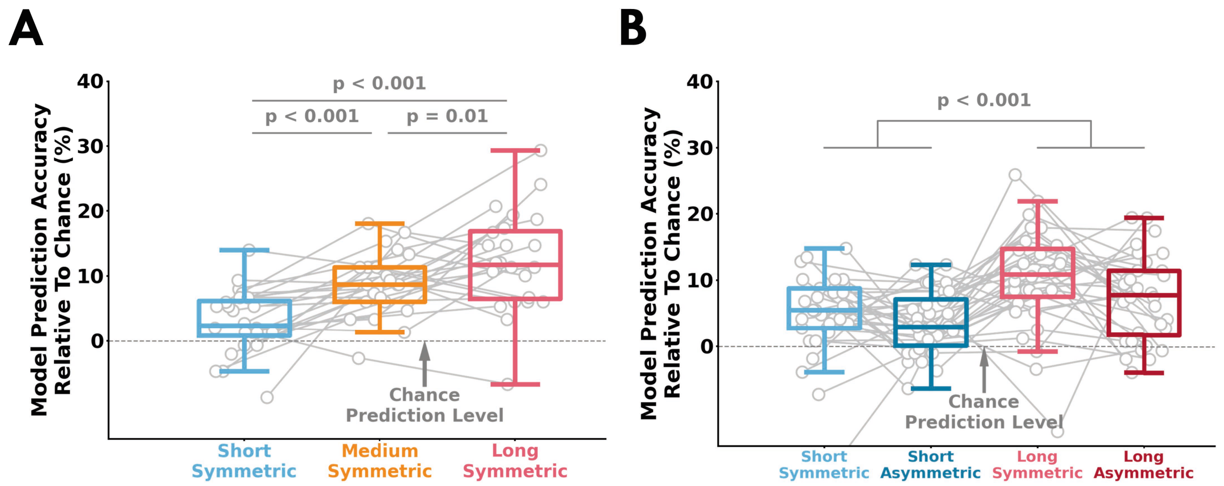

Figure S2: Model prediction accuracy. The *model prediction accuracy* (*y-axis*) of the drift-diffusion model considering wins, losses, and indecisions relative to chance for each condition in **(A)** Experiment 1 and **(B)** Experiment 2. The open gray circles and connecting gray lines correspond to individual participants. Box plots show 25th, 50th, and 75th percentiles. The *model prediction accuracy* was significantly greater than the chance level considering wins, losses and indecisions. Moreover, the *model prediction accuracy* increased with more available time due to utilizing accumulated sensory evidence.

Supplementary C

Leaky Accumulator Model

The leaky accumulator model is similar to the drift-diffusion model except that the accumulating evidence is allowed to leak over time. Sensory evidence was accumulated every 10 ms during the trial according to **Eq. 1**.

$$dE_{acc} = \mu E dt - l E_{acc} dt + \varepsilon \tag{1}$$

E_{acc} represents the accumulating sensory evidence. The parameter $\mu(0.007)$ represents the gain on the drift rate, and $l(0.001)$ controls the leak of accumulated sensory evidence. Gaussian noise (ε) was randomly sampled with a zero mean and standard deviation (σ) of 0.02. For consistency, we used the same sigmoidal bound described in the methods. Model prediction accuracy for the leaky accumulator model is shown on **Fig. S3A**.

Urgency-Gating Model

The urgency-gating model assumes that the sensory evidence is low-pass filtered and multiplied by a growing urgency signal, which is used to make a decision once it crosses a threshold. The decision variable, x_i, was calculated every 10 ms during the trial according to **Eq. 2**.

$$x_i = g E_f u + \varepsilon \tag{2}$$

Here, $g(8.0)$ represents a gain factor, E_f is the filtered sensory evidence, and u is the urgency signal. Gaussian noise (ε) was randomly sampled with a zero mean and standard deviation (σ) of 0.02. E_f was calculated by low-pass filtering the sensory evidence E with a time constant of τ (250 ms) as follows:

$$\tau \frac{dE_f}{dt} = -E_f + E \tag{3}$$

The urgency signal (u) was a linear function of trial time (t) normalized to the maximum available trial time (t_{max}; short = 500ms, medium = 850 ms, long = 1500 ms) as shown in **Eq. 4**.

$$u = \frac{t}{t_{max}} \tag{4}$$

For consistency, we used the same sigmoidal bound described in the methods. Model prediction accuracy for the urgency-gating model is shown on **Fig. S3B**.

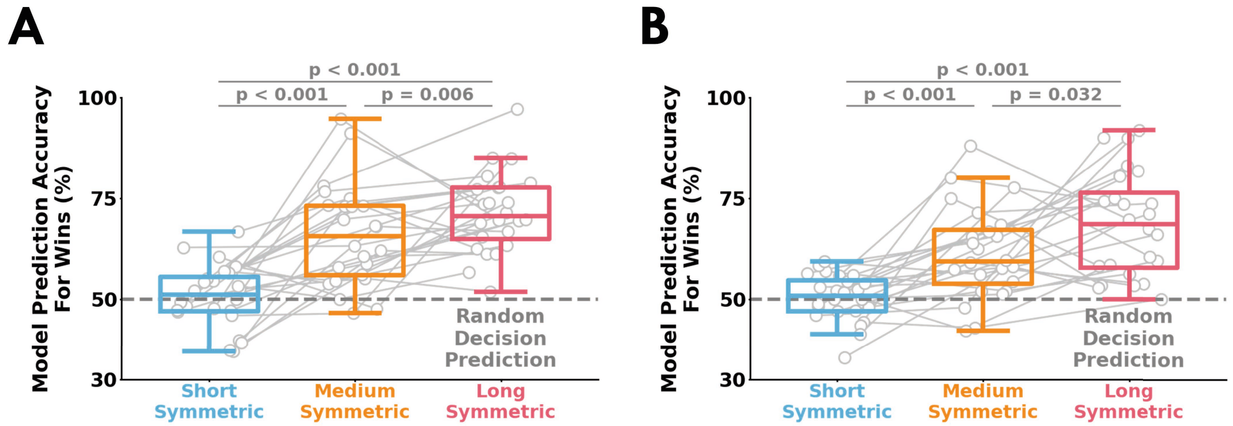

Figure S3: Model prediction accuracy. *Model prediction accuracy* (*y-axis*) for successful decisions as predicted by a **(A)** leaky accumulator model, and **(B)** urgency-gating model. The open gray circles and connecting gray lines correspond to individual participants. Box plots show 25th, 50th, and 75th percentiles.
